# MTBF-33: A multi-temporal building footprint dataset for 33 counties in the United States (1900 – 2015)

**DOI:** 10.1016/j.dib.2022.108369

**Published:** 2022-06-13

**Authors:** Johannes H. Uhl, Stefan Leyk

**Affiliations:** a University of Colorado Boulder, Cooperative Institute for Research in Environmental Sciences (CIRES) 216 UCB, Boulder, CO-80309, USA; b University of Colorado Boulder, Institute of Behavioral Science, 483 UCB, Boulder, CO-80309, USA; c University of Colorado Boulder, Department of Geography, 260 UCB, Boulder, CO-80309, USA

**Keywords:** Long-term land use change, Historical GIS, historical spatial data, Urbanization, Change detection, Remote sensing, Human settlements, Building stock, Built environment

## Abstract

Despite abundant data on the spatial distribution of contemporary human settlements, historical datasets on the long-term evolution of human settlements at fine spatial and temporal granularity are scarce, limiting our quantitative understanding of long-term changes of built-up areas. This is because commonly used large-scale mapping methods (e.g., computer vision) and suitable data sources (i.e., aerial imagery, remote sensing data, LiDAR data) have only been available in recent decades. However, there are alternative data sources such as cadastral records that are digitally available, containing relevant information such as building construction dates, allowing for an approximate, digital reconstruction of past building distributions. We conducted a non-exhaustive search of open and publicly available data resources from administrative institutions in the United States and gathered, integrated, and harmonized cadastral parcel data, tax assessment data, and building footprint data for 33 counties, wherever building footprint geometries and building construction year information was available. The result of this effort is a unique dataset that we call the Multi-Temporal Building Footprint Dataset for 33 U.S. Counties (MTBF-33). MTBF-33 contains over 6.2 million building footprints including their construction year, and can be used to derive retrospective depictions of built-up areas from 1900 to 2015, at fine spatial and temporal grain. Moreover, MTBF-33 can be employed for data validation purposes, or to train statistical learning models aiming to extract historical information on human settlements from remote sensing data, historical maps, or similar data sources.

## Specifications Table

**Table:** 

Subject	Geography
Specific subject area	Urban change detection, long-term land development, built environment, human settlements
Type of data	Geospatial vector data
How the data were acquired	Data were collected, integrated, and harmonized from web-based, open and publicly available sources published by local or regional governmental organizations, such as county or state governments.
Data format	Raw: ESRI Shapefile, ESRI File Geodatabase, Excel spreadsheets, CSV files. Filtered: ESRI Shapefile
Description of data collection	We identified U.S. counties or states that provide building footprint data and cadastral parcel data attributed with building construction year information. In a non-exhaustive search we identified 33 U.S. counties where these criteria were met. We integrated and harmonized these data to create geospatial vector datasets holding over 6.2 million building footprints attributed with their construction year.
Data source location	Source data was collected in 2016 from the following resources: ftp://ftp.co.ramsey.mn.us/GISdata/ (last accessed: 2016-03-01) ftp://ftp.lmic.state.mn.us/pub/data/elevation/lidar/county/ (last accessed: 2016-03-01) ftp://ftp1.fgdl.org/pub/state/ (last accessed: 2016-03-01) ftp://gisdata.co.anoka.mn.us/ (last accessed: 2016-03-01) http://bostonopendata.boston.opendata.arcgis.com/datasets/f3d274161b4a47aa9acf48d0d04cd5d4_3 (last accessed: 2016-03-01) http://data.evansvilleapc.opendata.arcgis.com/datasets/0f1a8007227641d394f4170acba8aa67_1 (last accessed: 2016-03-01) http://maps.co.mecklenburg.nc.us/openmapping/data.html (last accessed: 2022-03-02) http://opendata.arcgis.com/datasets/611eb2cad81a4089afa188233e6b6dd1_0 (last accessed: 2022-03-02) http://www.co.carver.mn.us/GIS (last accessed: 2022-03-02) http://www.co.dakota.mn.us/homeproperty/propertymaps/pages/default.aspx (last accessed: 2022-03-02) http://www.co.ramsey.mn.us/is/gisdata.htm (last accessed: 2016-03-01) http://www.co.washington.mn.us/index.aspx?NID=1606 (last accessed: 2022-03-02) https://city-tampa.opendata.arcgis.com/datasets/building-footprint (last accessed: 2017-12-01) https://data.cityofboston.gov/Permitting/Property-Assessment-2015/yv8c-t43q (last accessed: 2016-03-01) https://gisdata.mn.gov/dataset/us-mn-co-dakota-plan-parcels (last accessed: 2022-03-02) https://gisdata.mn.gov/dataset/us-mn-co-dakota-struc-propertyinfo-buildingp (last accessed: 2022-03-02) https://gisdata.mn.gov/dataset/us-mn-state-metrogis-plan-regonal-prcls-open (last accessed: 2016-03-01) https://gis-monmouthnj.opendata.arcgis.com/datasets/0d2bb2c31e854819939cae0e6e1b589b_0 (last accessed: 2018-12-02)
	https://gis-monmouthnj.opendata.arcgis.com/datasets/fec0b3d813174cdfb766134315120460_0/ (last accessed: 2022-03-02) https://koordinates.com/layer/102872-sarasota-county-florida-building-footprint/ (last accessed: 2022-03-02) https://mecklenburgcounty.exavault.com/share/view/mg5f-3uke3hyw (last accessed: 2022-03-02) https://opendata-bc-gis.hub.arcgis.com/datasets/building-footprints/ (last accessed: 2016-10-01) https://opendata-bc-gis.hub.arcgis.com/datasets/parcels/explore (last accessed: 2022-03-02) https://public-manateegis.opendata.arcgis.com/datasets/building-footprints (last accessed: 2017-07-01) https://www.bouldercounty.org/government/open-data/ (last accessed: 2022-03-02) https://www.bouldercounty.org/government/open-data/) (last accessed: 2022-03-02) https://www.mass.gov/get-massgis-data (last accessed: 2022-03-02)
Data accessibility	Repository name: Mendeley Data Data identification number: 10.17632/w33vbvjtdy Direct URL to data: https://data.mendeley.com/datasets/w33vbvjtdy
Related research article	S. Leyk, J. H. Uhl, D. Balk, B. Jones, Assessing the accuracy of multi-temporal built-up land layers across rural-urban trajectories in the United States, Remote Sens. Environ. 204 (2018). https://doi.org/10.1016/j.rse.2017.08.035.

## Value of the Data

• Open and publicly accessible data on building age are scarce. Our data scraping, integration, and harmonization effort aims to fill this gap in the data landscape.
• Knowledge of the construction year of individual buildings allows for creating spatially and temporally fine-grained depictions of past built-up surfaces.
• Such spatial-historical data may serve as calibration and validation data for urban change models and for historical (and more recent) human settlement datasets, as well as for historical population downscaling efforts.
• Moreover, such data are very useful to be employed as auxiliary data for the automated training data generation for data-intensive (e.g. deep learning) computer vision models to automatically extract urban change signals from remote sensing data or historical maps.
• These data enable historical analyses of the building stock in 33 U.S. counties, encompassing the whole state of Massachusetts, as well as several urban areas of different settlement age and characteristics, such as Boston, Charlotte, and Minneapolis.
• Lastly, these data are highly valuable for urban planners, remote sensing analysts, historians, demographers, and data scientists working in the context of urban land use change and (sub)urbanization, as they provide rare insight into the long-term dynamics of built-up areas at very high spatial and temporal detail.

## 1 Data Description

**Fig. 1:**
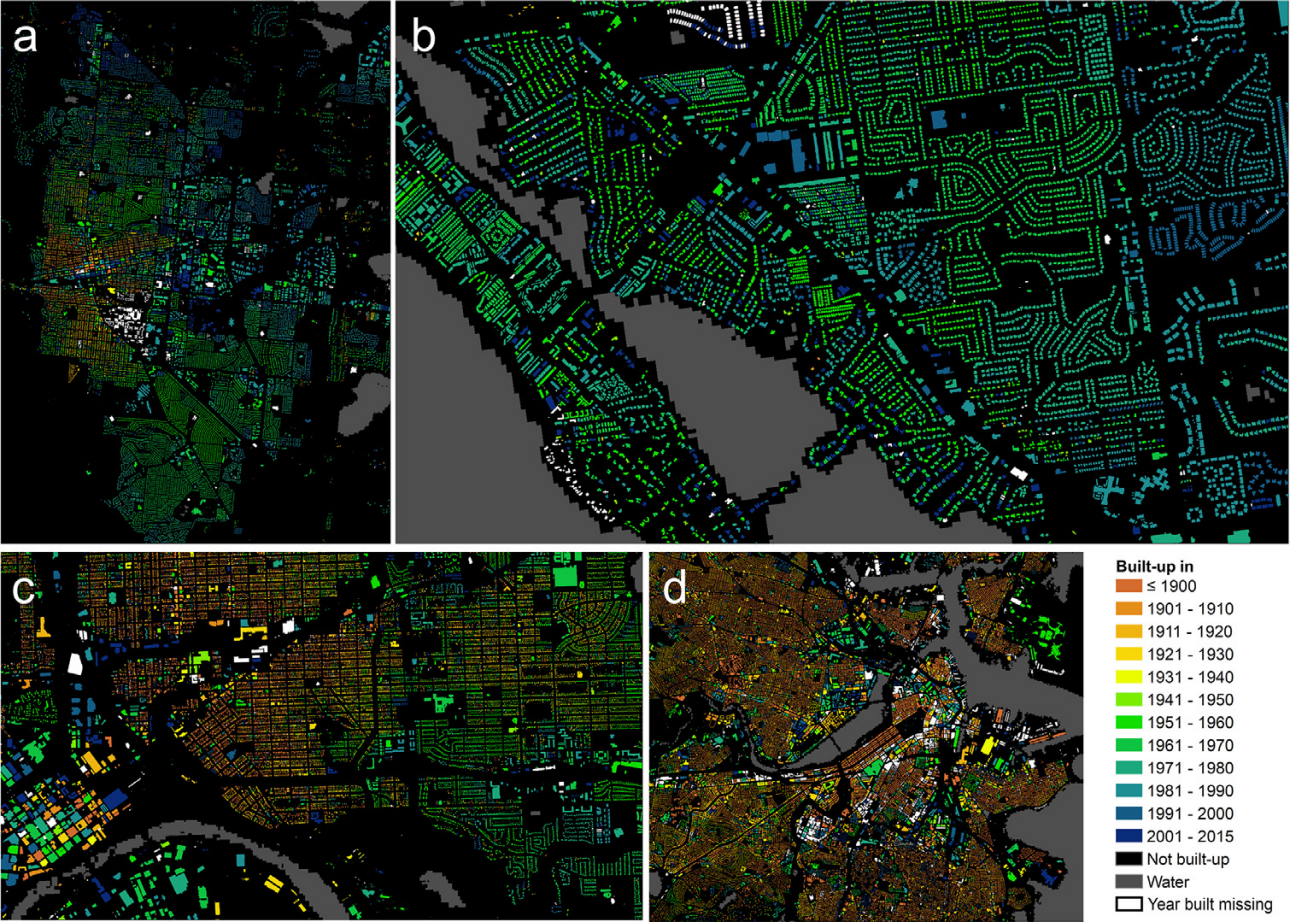
MTBF-33 multi-temporal building footprint data examples, shown for (a) Boulder (Colorado) (b) Sarasota (Florida), (c) Boston (Massachusetts), and (d) Minneapolis (Minnesota).

We collected open and publicly available data resources from the web from administrative, county- or state-level institutions in the United States and integrated and harmonized cadastral parcel data, tax assessment data, and building footprint data for 33 counties, where building footprint data and building construction year information (“year built”) was available. The result of this effort is a unique dataset called the Multi-Temporal Building Footprint Dataset for 33 U.S. Counties (MTBF-33, [1], available at http://dx.doi.org/10.17632/w33vbvjtdy). MTBF-33 contains over 6.2 million building footprints including their construction year, and is available as polygonal geospatial vector data in 33 ESRI Shapefiles, in geographic coordinates (WGS84, EPSG:4326), as well as projected into Albers equal area conic projection for the contiguous USA (USGS version, SR-ORG:7480^1^), organized per county. Fig. 1 shows small subsets of the MTBF-33 dataset for selected regions.

**Fig. 2:**
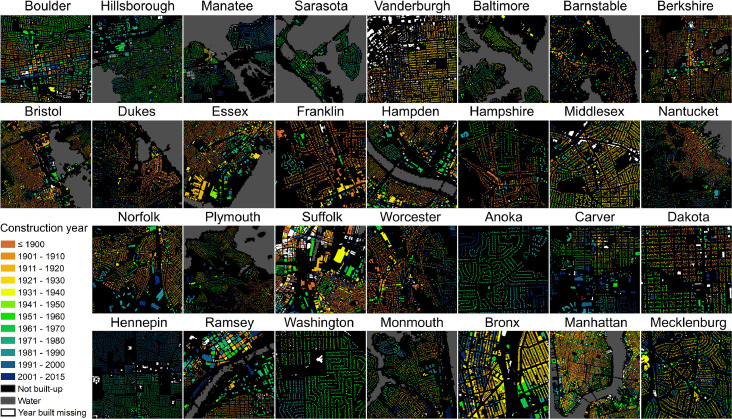
MTBF-33 multi-temporal building footprint dataset, showing examples for most of the 33 counties covered in MTBF-33. Water mask in grey derived from GHS-BUILT R2018A (epoch 2014, [2]). Counties are sorted by their FIPS in the same order as shown in Table 1 (upper left: Boulder County, lower right: Mecklenburg county). Parts of New York County and Kings County (New York City) are jointly shown as “Manhattan”. Not shown are Queens and **Richmond counties (New York City)**.

Moreover, Fig. 2 shows small subsets of the data for most of the 33 U.S. counties, illustrating the variability in the data and their coverage across different geographic settings.

As can be seen in Figs. 1 and 2, there are several buildings without year built attribute (white color). We report the year built attribute completeness for each of the 33 counties in Table 1. Moreover, Table 1 shows some basic year built statistics, illustrating the variety in temporal coverage of the data.

**Table 1:** Overview and statistics of the 33 counties covered in MTBF-33.

County	County		Buildings w/	Total	Percent	Year built	Year built	Year built	Year built
FIPS	Name	State	valid year built	buildings	complete	minimum	maximum	mean	median
08013	Boulder	Colorado	76,929	80,255	95.9	1858	2014	1968	1971
12057	Hillsborough	Florida	410,076	421,046	97.4	1842	2014	1981	1984
12081	Manatee	Florida	154,416	173,173	89.2	1870	2013	1980	1982
12115	Sarasota	Florida	194,043	198,685	97.7	1877	2013	1981	1981
18163	Vanderburgh	Indiana	93,797	108,798	86.2	1810	2015	1951	1950
24005	Baltimore	Maryland	281,216	308,933	91.0	1676	2015	1958	1958
25001	Barnstable	Massachusetts	172,542	185,818	92.9	1626	2014	1961	1971
25003	Berkshire	Massachusetts	82,036	89,790	91.4	1650	2015	1937	1950
25005	Bristol	Massachusetts	225,156	233,704	96.3	1500	2013	1948	1957
25007	Dukes	Massachusetts	18,758	23,524	79.7	1660	2014	1963	1980
25009	Essex	Massachusetts	224,351	270,398	83.0	1600	2014	1937	1947
25011	Franklin	Massachusetts	43,436	50,209	86.5	1666	2013	1936	1951
25013	Hampden	Massachusetts	192,281	207,195	92.8	1600	2015	1947	1953
25015	Hampshire	Massachusetts	69,505	77,982	89.1	1629	2014	1947	1960
25017	Middlesex	Massachusetts	460,722	500,047	92.1	1600	2015	1942	1950
25019	Nantucket	Massachusetts	13,547	13,971	97.0	1640	2011	1962	1983
25021	Norfolk	Massachusetts	216,150	242,631	89.1	1500	2015	1944	1951
25023	Plymouth	Massachusetts	207,264	230,788	89.8	1600	2015	1950	1962
25025	Suffolk	Massachusetts	106,037	109,876	96.5	1637	2015	1924	1920
25027	Worcester	Massachusetts	317,302	344,307	92.2	1650	2015	1948	1957
27003	Anoka	Minnesota	128,498	135,307	95.0	1852	2015	1977	1979
27019	Carver	Minnesota	40,488	41,768	96.9	1816	2015	1969	1984
27037	Dakota	Minnesota	145,903	163,179	89.4	1832	2014	1973	1978
27053	Hennepin	Minnesota	380,301	387,856	98.1	1843	2010	1955	1956
27123	Ramsey	Minnesota	239,544	245,279	97.7	1850	2015	1946	1951
27163	Washington	Minnesota	86,216	95,014	90.7	1742	2015	1973	1983
34025	Monmouth	New Jersey	206,624	212,951	97.0	1684	2015	1961	1963
36005	Bronx	New York	102,658	103,865	98.8	1780	2015	1941	1931
36047	Kings	New York	329,283	331,813	99.2	1800	2015	1931	1925
36061	New York	New York	45,322	46,209	98.1	1765	2014	1921	1910
36081	Queens	New York	454,506	457,628	99.3	1661	2015	1939	1935
36085	Richmond	New York	138,609	140,050	99.0	1665	2014	1962	1969
37119	Mecklenburg	North Carolina	402,242	418,056	96.2	1792	2015	1980	1984

## 2 Experimental Design, Materials and Methods

1) Data creation

We manually collected cadastral parcel data, tax assessment data, and building footprint data from publicly and openly available web resources, such as from state-level or county-level administrative GIS or spatial data resources. We used open data portals such as https://hub.arcgis.com to identify counties or states where (a) both parcel and building footprint data is available, and (b) parcel data or joinable tax assessment data contains information on the year when structures have been established (year built). We identified 33 counties that satisfied these criteria and where the completeness of the building footprint data and the year built attribute was acceptable (see Table 1).

In counties where the year built information was contained in separate tax assessment datasets, we first joined the tax assessment data to the parcel data. Then, we integrated the parcel data and building footprint data. This integration was done through a spatial join operation, in order to transfer the year built attribute from the parcel polygon features to the building footprint features contained within the cadastral parcel boundaries. This spatial assignment was based on a majority-area criterion in order to account for certain levels of spatial offsets between parcel and building footprint data. Such offsets may exist due to different data acquisition methods: While parcel boundaries are typically measures using terrestrial or Global Navigation Satellite System (GNSS)-based land surveying technologies, building footprint data may be obtained through automatic segmentation of LiDAR data or by digitization in aerial imagery.

As a result of these spatial joins, the year built attribute was transferred to the building footprint features. For these processes, we used the GeoPandas^2^ and ESRI ArcPy^3^ python package. We then harmonized and cleaned the data. This cleaning process involved the identification of non-plausible year built values (e.g., < 1500). We do this to remove structural zero values representing missing information, and obviously incorrect values, likely resulting from typos, such as “910” which could be “1910”. The threshold of 1500 was chosen as it marks the approximate end of the Pre-Columbian era, and very few built-up structures or dwellings from that era are still intact, and if so, they are rather considered a monument or landmark than a “building” as defined in modern building stock databases. Such missing or non-plausible year built values were set to 0. Importantly, any property-, building-, or individual-level data other than the year built attribute was removed, so that the MTBF-33 data exclusively consists of building footprint geometries and their construction year. The resulting polygonal, geospatial vector data represent building footprints for 33 counties in the conterminous United States, allowing for the reconstruction of spatially and temporally fine-grained depictions of built-up surfaces (i.e., building level, annual temporal resolution).

While contemporary building footprint data is available at high levels of accuracy [3], data on the historical distributions of the U.S. building stock is very scarce, in particular for time periods earlier than the 1970s or 1980s, when remote-sensing-based, digital earth observation data became accessible. Thus, despite representing only about 1% of the U.S. counties, this unique dataset covers more than 40,000 km² and more than 6,000,000 cadastral parcels, and fills an important gap in the geospatial data landscape. The MTBF-33 dataset was collected in 2016-2017 and since then, MTBF-33 has been employed by the authors for different purposes, including the validation of global remote-sensing-based multi-temporal built-up surface data [4], [5], [6], [7], [8], the validation of historical settlement data derived from property databases [9,10], to automatically generate training data for urban change detection based on Landsat time series data [11], to assess the sensitivity of Landsat time series to urban changes [12], and for training data generation used by computer vision models to extract settlement patterns from historical topographic maps [13,14].

2) Validation and uncertainty assessment:

The validation of data that entail advancements in quality and accuracy compared to existing data products is always challenging. We evaluated MTBF-33 through two approaches. First, we compared the dataset for all 33 counties with the Microsoft Building Footprint (MSBF) dataset [3] for the building footprints existing in 2015. Second, we carried out a visual comparison between the MTBF-33 and urban extents as found in historical topographic maps.

### 2.1 Agreement assessment between MTBF-33 and Microsoft building footprint data

Microsoft's building footprint dataset (data release from 2018) has a US-wide coverage and has been extracted from Microsoft Bing imagery using a deep-learning-based computer vision method. While the acquisition years of the Bing imagery are likely to vary across the United States, we assume that MSBF represents the U.S. building stock approximately in 2015, expecting an average period of three years between image data acquisition and building footprint data release. In order to evaluate the MTBF-33 quantitatively, we created gridded binary layers (i.e., built-up versus not built-up) for 2015 for both MTBF-33 and MSBF, in a spatial grid of 250m x 250m, based on an area majority rule, for each of the 33 counties covered in MTBF-33. Based on these gridded surfaces, we established confusion matrices per county, used to calculate various agreement measures to assess agreement between the two binary layers, using the MSBF data as reference data (Table 2). While some of these measures have been criticized due to some limitations, e.g., if class proportions are imbalanced [15,16], a cross-section through all those measures represents a reliable assessment basis. We carried out the agreement assessment across all 33 counties, as well as separately for higher-density and lower-density counties. This stratification was done based on the MSBF built-up surface density per county, using the median county-level built-up surface density as a threshold.

**Table 2:** Results of the agreement assessment between MTBF-33 (in 2015) and Microsoft building footprint data.

Agreement measure	All counties	Higher-density counties	Lower-density counties
Overall accuracy	0.933	0.969	0.919
Precision	0.956	0.960	0.954
Recall	0.900	0.990	0.853
F-measure	0.928	0.974	0.901
Kappa index	0.865	0.935	0.833

As can be seen in Table 2, when using all 33 counties for map comparison, all accuracy measures show high agreement between the two layers (between 86.5% based on Kappa and 95.6% based on Precision). Higher accuracy is observed for high-density counties compared to the low-density counties. The notable difference in Recall (0.99 and 0.85, respectively) indicates that omission errors are higher in low-density counties, possibly because MSBF identifies structures that are not part of the county assessor's building stock database (e.g., barns), especially in more rural settings. Thus, MTBF-33 has no built structures at those locations, resulting in higher proportions of false negatives. This effect propagates into the other measures, resulting in reduced F-measure and Kappa index for lower-density counties. However, even in lower-density counties, no accuracy measure is less than 0.83 indicating high levels of agreement between the two datasets. Moreover, there may be slight temporal gaps between MTBF-33 (representing the building stock in 2015) and MSBF (heterogeneous acquisition years of the imagery underlying the MSBF data), due to the heterogeneous levels of temporal coverage of the MTBF-33 data (see built year statistics in Table 1), and due to the vagueness in the definition of the construction year in MTBF-33. These factors could explain some of the slight disagreement observed in Table 2. Here, it is worth noting that the aforementioned, assumed period of three years between Bing imagery acquisition and data release in 2018 may be overly optimistic, i.e., the underlying imagery may have been acquired prior to 2015 and thus, MSBF may reflect an earlier state of the building stock. Assuming predominant growth (rather than shrinkage) of the building stock over time, such an incorrect time stamp of our reference data (i.e., MSBF) would result in artificially inflated commission errors (i.e., low precision) in our test data (i.e., the MTBF-33) which represents a later state of the building stock. However, as shown in Table 2, Precision is very high across all strata, and thus, the unknown temporal reference of the MSBF data and the effects of a potential temporal gap may explain the observed commission errors of 0.040 to 0.046.

### 2.2 Qualitative evaluation of MTBF-33 data against historical maps

**Fig. 3:**
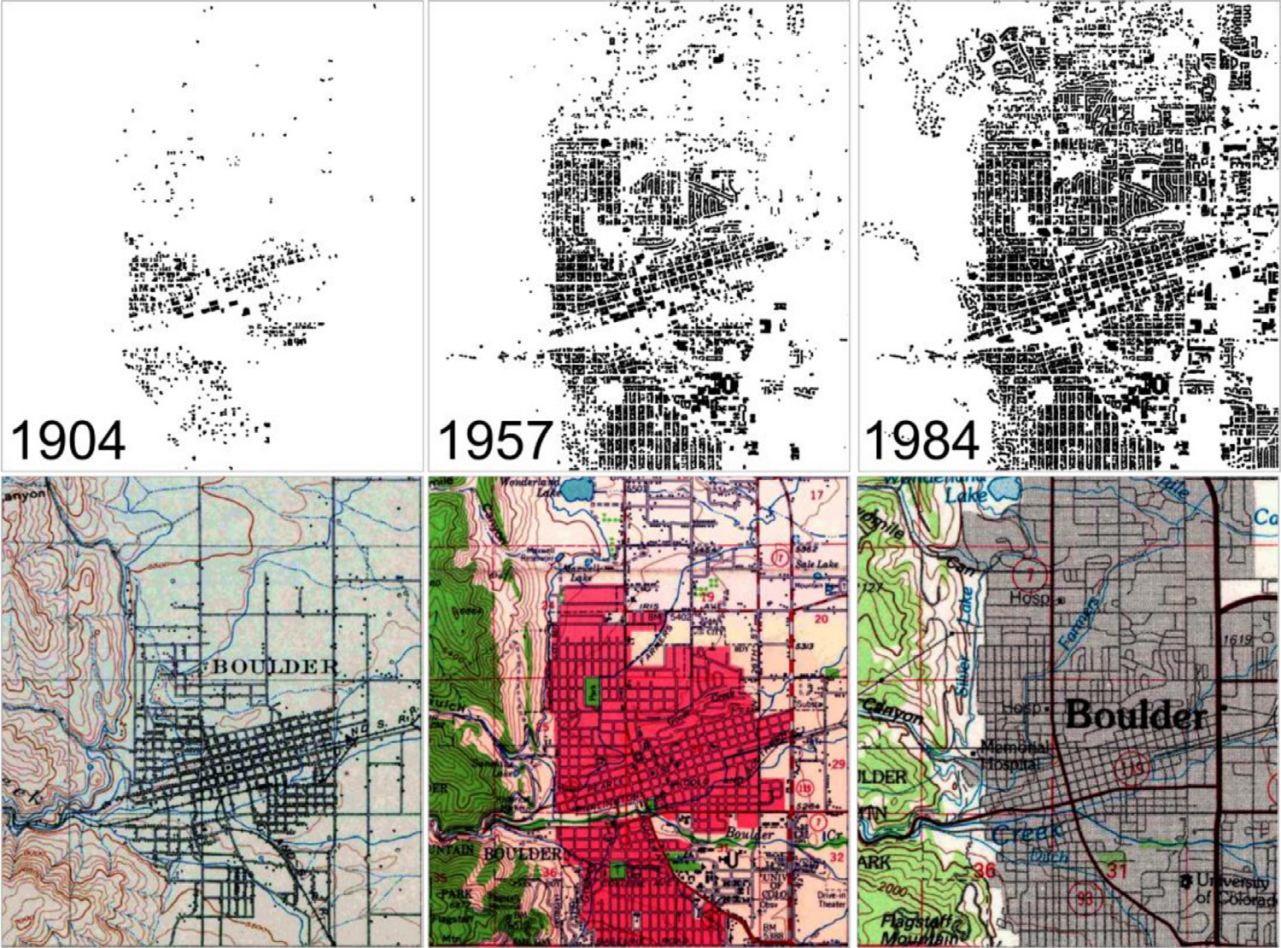
Comparison of retrospective building footprint distributions to historical maps for Boulder, Colorado, in 1904 (scale 1:62,500), 1957 (scale 1:62,500), and 1984 (scale 1:100,000). Map source: USGS-HTMC.

We carried out a visual comparison between the MTBF-33 and urbanized extents as shown in historical topographic maps of the U.S. Geological Survey (USGS) historical topographic map collection (HTMC^4^) [17]. To do so, we created historical binary layers of the MTBF-33 to match the publication dates of the historical map sheets. Fig. 3 shows historical map sheets for Boulder, Colorado, and the respective extracted MTBF-33 binary layers for 1904, 1957 and 1984. As can be seen, the spatial representations of built-up / urbanized areas generally agree. Agreement is highest within urban areas as shown by denser building blocks along roads in 1904, in red in 1957 and grey in 1984, even though MTBF-33 shows much finer spatial detail of the built-up areas. Outside the urbanized areas, the 1904 and 1957 maps show detailed building symbols along roads, many of which are also visible in the MTBF-33 layer. Some discrepancies can be seen in the North-west part of the 1984 map, where some buildings are visible in MTBF-33 but not in the historical map. This is due to the level of cartographic generalization used in the 1984 map sheet (scale 1:100,000, whereas the 1904 and 1957 maps are at scale 1:62,500) which may not include individual building footprints outside urban extents.

Moreover, such discrepancies may be due to temporal uncertainty in the historical maps (i.e., temporal gap between land surveying or field check, and map edition / publishing year) and due to the potential uncertainty of the construction year information in MTBF-33. It is unknown whether the date on record reflects the beginning or the end of the building construction phase, and how long the construction phase endured. Moreover, the construction year could be an estimate, and buildings may be missing in MTBF-33 because of incomplete records or missing built year information. However, the visual similarity for the two historical map sheets combined with the quantitative agreement assessment against Microsoft's building footprint dataset provide strong confidence that the built-up surfaces in MTBF-33 are very plausible and accurate with some local variations in completeness.

There are uncertainties in MTBF-33 that are very difficult to measure. For example, buildings that have been torn down or were destroyed are not included in the dataset. The respective parcels may have become vacant land or a new structure may have been built. As a consequence, there is survivorship bias in construction year information which increases as we go back in time. There are few studies that report on or measure survivorship bias in settlement layers as this requires access to historical versions of cadastral data or demolition records (e.g., [18], [19], [20], [21]. For example, McShane et al. [21] used historical demolition data for Colorado and found that survivorship bias had limited impact on the resulting settlement layers, resulting in relative errors of less than 2%. Note that while we retain all plausible year built values from the scraped source data, we constrain the temporal coverage to the period 1900 – 2015. We discourage data users to use MTBF-33 to create snapshots of built-up surfaces preceding the year 1900, as the survivorship bias may be very large.

## Ethics Statements

None.

## CRediT Author Statement

**Johannes H. Uhl:** Methodology, Investigation, Data curation, Validation, Visualization, Writing - Original Draft; **Stefan Leyk:** Conceptualization, supervision, writing - reviewing and editing.

## Declaration of Competing Interest

The authors declare that they have no known competing financial interests or personal relationships that could have appeared to influence the work reported in this paper.

## Data Availability

MTBF-33: A multi-temporal building footprint dataset for 33 U.S. counties at annual resolution (1900-2015) (Original data) (Mendeley Data).

## Footnotes

1 https://spatialreference.org/ref/sr-org/7480/

2 https://geopandas.org

3 https://www.esri.com/pythonlibraries

4 Available at https://ngmdb.usgs.gov/topoview/

## References

[1] Uhl J.H., Leyk S. (2022). MTBF-33: A multi-temporal building footprint dataset for 33 U.S. counties at annual resolution (1900-2015). https://data.mendeley.com/datasets/w33vbvjtdy.

[2] Florczyk A.J., Corbane C., Ehrlich D., Freire S., Kemper T., Maffenini L., Melchiorri M., Pesaresi M., Politis P., Schiavina F. Sabo M. (2019). GHSL data package 2019. Luxemb. EUR.

[3] Microsoft (2018). https://github.com/microsoft/USBuildingFootprints.

[4] Uhl J.H., Leyk S., Florczyk A.J., Pesaresi M., Balk D. (2016). Int. Conf. GIScience Short Pap. Proc.

[5] Uhl J.H., Zoraghein H., Leyk S., Balk D., Corbane C., Syrris V., Florczyk A.J. (2018). Exposing the urban continuum: Implications and cross-comparison from an interdisciplinary perspective. Int. J. Digit. earth.

[6] Leyk S., Uhl J.H., Balk D., Jones B. (2018). Assessing the accuracy of multi-temporal built-up land layers across rural-urban trajectories in the United States. Remote Sens. Environ..

[7] J.H. Uhl, S. Leyk, A framework for scale-sensitive, spatially explicit accuracy assessment of binary built-up surface layers, arXiv Prepr. arXiv2203.11253 (2022), doi:10.48550/arXiv.2203.11253.

[8] J.H. Uhl, S. Leyk, Uncertainty prediction of built-up areas from global human settlement data in the United States based on landscape metrics. arXiv Prepr arXiv2205.09023 (2022), doi:10.48550/arXiv.2205.09023.

[9] Leyk S., Uhl J.H. (2018). HISDAC-US, historical settlement data compilation for the conterminous United States over 200 years. Sci. data.

[10] Uhl J.H., Leyk S., McShane C.M., Braswell A.E., Connor D.S., Balk D. (2021). Fine-grained, spatiotemporal datasets measuring 200 years of land development in the United States. Earth Syst. Sci. data.

[11] Uhl J.H., Leyk S. (2020). Towards a novel backdating strategy for creating built-up land time series data using contemporary spatial constraints. Remote Sens. Environ..

[12] J.H. Uhl, S. Leyk, A framework for radiometric sensitivity evaluation of medium resolution remote sensing time series data to built-up land cover change, Int. Geosci. Remote Sens. Symp., 2017, doi:10.1109/IGARSS.2017.8127351.

[13] Uhl J.H., Leyk S., Chiang Y.Y., Duan W., Knoblock C.A. (2017).

[14] Uhl J.H., Leyk S., Chiang Y.Y., Duan W., Knoblock C.A. (2018). Spatialising uncertainty in image segmentation using weakly supervised convolutional neural networks: a case study from historical map processing. IET Image Process.

[15] Pontius R.G., Millones M. (2011). Death to Kappa: birth of quantity disagreement and allocation disagreement for accuracy assessment. Int. J. Remote Sens..

[16] Stehman S.V., Wickham J. (2020). A guide for evaluating and reporting map data quality: Affirming Shao et al.“Overselling overall map accuracy misinforms about research reliability. Landsc. Ecol..

[17] G.J. Allord, J.L. Walter, K.A. Fishburn, G.A. Shea, Specification for the US Geological Survey Historical Topographic Map Collection. U.S. Geological Survey Techniques and Methods, book 6, chap. B11, 65 p., doi:10.3133/tm11B6.

[18] Tanikawa H., Hashimoto S. (2009). Urban stock over time: spatial material stock analysis using 4d-GIS. Build. Res. Inf..

[19] Aksözen M., Hassler U., Kohler N. (2017). Reconstitution of the dynamics of an urban building stock. Build. Res. Inf..

[20] Aksözen M., Hassler U., Rivallain M., Kohler N. (2017). Mortality analysis of an urban building stock. Build. Res. Inf..

[21] C.M. McShane, J.H. Uhl, S. Leyk, Gridded land use data for the conterminous United States 1940-2015. Scientific Data (accepted). 10.1038/s41597-022-01591-0 PMC9376068 35963932

